# Predictive Role of Serum Inflammatory Markers in Metastatic Luminal A/B Breast Cancers Receiving CDK‐4/6 Inhibitors Therapy

**DOI:** 10.1002/iid3.70442

**Published:** 2026-04-22

**Authors:** Nilufer Bulut, Tanju Kapagan, Aykut Ozmen, Gokmen Umut Erdem

**Affiliations:** ^1^ Department of Medical Oncology University of Health Sciences Istanbul Turkey; ^2^ Department of Medical Oncology Basaksehir Cam and Sakura City Hospital Istanbul Turkey

**Keywords:** breast cancer, CDK‐4/6i, metastasis, neutrophil and lymphocyte ratio

## Abstract

**Background:**

We conducted this study to evaluate the impact of serum inflammatory markers and histopathological features on prognosis in metastatic hormone receptor positive breast cancer patients treated with first line CDK4/6i (cyclin‐dependent kinase inhibitors).

**Objectives:**

While serum markers are better indicators in triple‐negative and HER‐2 positive breast cancers, the PFS (progression‐free survival) durations of NLR (neutrophil/lymphocyte ratio), PLR (platelet/lymphocyte ratio), and LMR (platelet/lymphocyte ratios) in luminal A–B group patients vary between studies.

In our study investigated whether baseline NLR, PLR, and LMR have a predictive value for PFS in hormone‐positive metastatic breast cancer receiving CDK4/6i.

**Material and Methods:**

The study included 111 patients with de novo metastasis or recurrence/metastasis after adjuvant/neo‐adjuvant treatment who received CDK‐4/6i as first‐line therapy. NLR, PLR, LMR, lymphocyte, neutrophil, and monocyte values were recorded from peripheral blood analyses before CDK‐4/6i treatment.

**Results:**

When the patient characteristics of patients were analyzed for progression‐free survival, no statistically significant differences were found between age, ECOG, grade, Ki‐67 index, and menopause status. PFS was not significantly associated with NLR, PLR, and LMR values (*p* = 0.87, *p* = 0.51, *p* = 0.22, respectively. In univariate analysis, among patients with LMR > 3.48, ribociclib was associated with longer PFS than palbociclib. Multivariate analysis revealed that the luminal B molecular subtype was associated with significantly worse PFS compared to luminal A (23 vs. 43 months, respectively, *p* = 0.01).

**Conclusions:**

The prognostic impact of serum inflammatory markers in hormone receptor–positive metastatic breast cancer is heterogeneous. Although no statistical significance was observed in our cohort, high LMR levels may provide predictive and prognostic value in treatment selection.

## 1 Introduction

Cyclin‐dependent kinase 4/6 (CDK4/6i) inhibitors are used to treat de novo or recurrent metastatic disease in pre‐ or post‐menopausal women with primary or secondary endocrine resistance. CDK4/6i are often used in combination with aromatase inhibitors and/or luteinizing hormone‐releasing hormone agonists; they may also be used in combination with fulvestrant. Compared with chemotherapeutic agents, CDK4/6i therapy provides better quality of life, longer progression‐free survival (PFS), and longer overall survival (OS) [1]. However, no predictive markers are available for determining treatment responses to these agents.

The neutrophil/lymphocyte ratio (NLR), platelet/lymphocyte ratio (PLR), and lymphocyte/monocyte ratio (LMR) in peripheral blood are used as markers of chronic inflammation, especially in patients with triple‐negative breast cancers, to assess their responses to neoadjuvant treatment and predict their prognoses [2]. However, data regarding the associations of these markers with treatment response and prognosis in patients with hormone receptor (HR)‐positive metastatic breast cancer are limited [3].

## 2 Objectives

This study was performed to determine the ability of the baseline NLR, PLR, and LMR in peripheral blood to predict the treatment response and progression‐free survival in patients with metastatic HR‐positive breast cancer receiving first‐line treatment with CDK4/6 inhibitors.

## 3 Materials and Methods

### 3.1 Study Participants

This retrospective analysis involved 111 patients with estrogen receptor (ER)‐positive, progesterone receptor (PR)‐positive, human epidermal growth factor receptor 2 (HER2)‐negative metastatic breast cancer who were treated from February 1997 to July 2024. These patients had received first‐line CDK4/6i therapy in the metastatic setting. Palbociclib or ribociclib was combined with an aromatase inhibitor or fulvestrant. CDK4/6 inhibitors were used for 21 days with a 1‐week break. Oral anti‐estrogen therapy was continued daily throughout treatment. Fulvestrant was administered once a month after an initial 15‐day loading period.

*The inclusion criteria* for this study were a diagnosis of HR‐positive, HER2‐negative de novo metastatic breast cancer, as well as primary and secondary endocrine‐resistant patients using first‐line CDK4/6 inhibitors. HR‐positivity was defined as a receptor expression of 10% or higher. Primary endocrine resistance was defined as recurrence or metastasis occurring within the first 2 years while receiving adjuvant endocrine therapy. Secondary endocrine resistance was defined as a relapse occurring after at least 2 years of endocrine therapy, either during the treatment or within the first year after completing adjuvant endocrine therapy.

*The exclusion criteria* were autoimmune disease, granulomatous disease, or acute or chronic inflammatory disease; pregnancy; use of filgrastim before CDK4/6 inhibitor therapy; insufficient data; and receipt of CDK4/6 inhibitor therapy at any stage of treatment with proven progression by radiological imaging under multiple chemotherapy (or hormonal treatment due to lack of reimbursement) in the metastatic setting.

### 3.2 Data Collection

The following patient data were recorded: age, Scarff–Bloom–Richardson grade [4], Eastern Cooperative Oncology Group (ECOG) performance status [5], TNM stage, histological diagnosis, hormone receptor status, de novo or secondary metastasis status, treatment received, metastatic sites, initiation and termination dates of CDK4/6 inhibitor treatment. Other variables included NLR, PLR, lymphocyte count, neutrophil count, platelet count, monocyte count, treatment response, and date of last visit.

Blood tests were performed 1 month before initiation of treatment with CDK4/6 inhibitors. Blood tests were also conducted prior to treatment to predict host and tumor response (the best tumor response in the first 3 months). Continuous blood testing during CDK4/6i treatment would typically normalize these values. Baseline inflammatory cell counts were determined before treatment to reflect tumor immunity [6].

**Figure 1:**
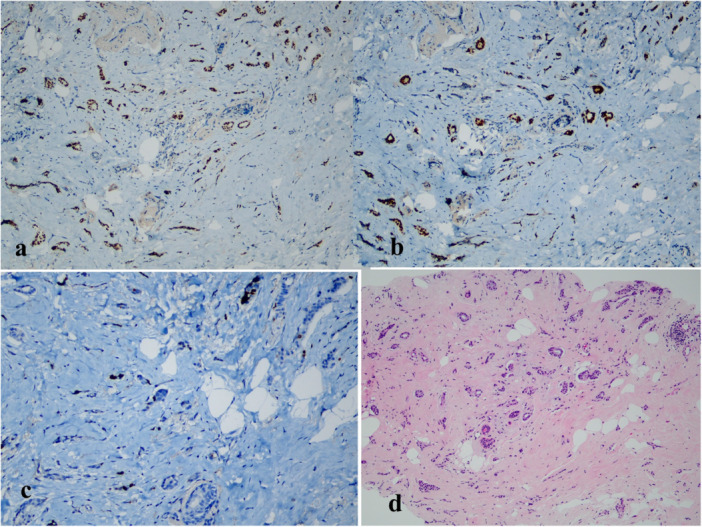
Luminal A; Representing staining results from H&E and IHC for ER, PR, and Ki‐67 (20 × magnification). ER and PR positive required at least 10% staining nuclei (a–b), Ki‐67 ≤ 20% (c), invasive ductal carcinoma NOS (d).

**Figure 2:**
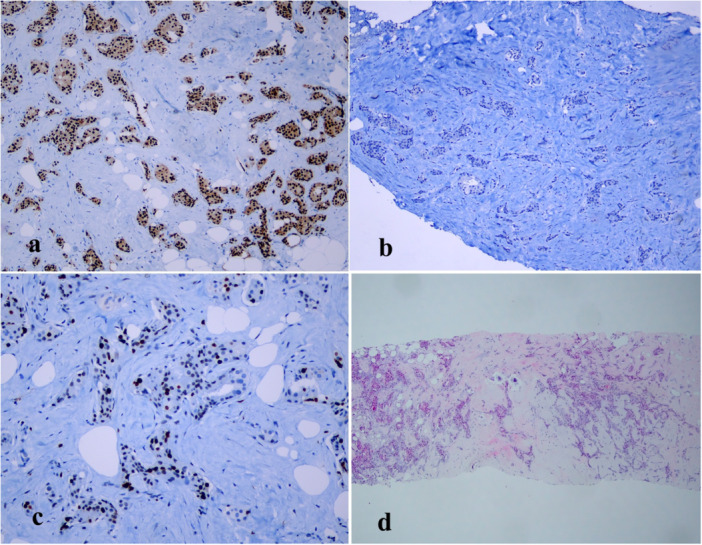
Luminal B; Representing staining results from H&E and IHC for ER positive required at least 10% staining nuclei (a) (20 × magnification), PR negative (b), Ki‐67 > 20% (c), invasive ductal carcinoma NOS (d).

The NLR, PLR, and LMR were calculated. Lymphopenia was defined as an absolute lymphocyte count of < 1.5 G/L. Immunohistochemistry was used to determine ER, PR, and HER2 statuses. ER and PR were stained by using clones DAKO and scored by the Allred scoring method [7]. Chromogenic in situ hybridization was performed for HER2+ cases. Cancers were divided into two subgroups based on histopathologic features: luminal A (ER +, PR +, HER2 −, Ki‐67 ≤ 20%) and luminal B (ER +, PR ±, HER2‐, Ki‐67 > 20%) (Figures 1, 2).

Hormone‐positive tumors (HR) generally show limited benefit from endocrine therapy, and HR‐positivity was defined as 10% or higher [8]. While selective estrogen modulators (SERMs) or selective estrogen degraders (SERDs) were preferred, patients with ER‐positivity 10% or above were selected for hormone sensitivity [9].

The NLR, PLR, and LMR were analyzed using high and low cut‐off values. PFS was defined as the time from initiation of CDK4/6 inhibitor therapy to the diagnosis of progressive disease, according to the Response Evaluation Criteria in Solid Tumors (RECIST) Version 1.1 [10]. Treatment response was evaluated by computed tomography and positron emission tomography/computed tomography (PET/CT) after three cycles of treatment. Responses were recorded as complete response, partial response, stable disease, or progressive disease, according to the RECIST criteria. Univariate and multivariate analyses were performed to assess the impact of NLR, PLR, and LMR on treatment outcomes.

### 3.3 Statistical Methods

Statistical analyses were performed using PASW Statistics software (version 22.0; SPSS Inc., Chicago, IL, USA). Descriptive statistics were reported using the median for non‐normally distributed data. S Categorical variables are expressed as frequencies (*n*) with percentages (%). Categorical variables were analyzed using the Chi‐squared test or Fisher's exact test. The Mann–Whitney *U*‐test was used to compare non‐normally distributed variables of two groups. Survival curves were estimated by the Kaplan–Meier method. The factors identified in the univariate analysis (*p* < 0.15) were entered into the Cox regression analysis with backward selection to determine independent predictors of survival. Variables with a *p* value < 0.05, and variables with a clinically significant *p* value < 0.15 were analyzed in multivariate analysis. Progression‐free survival (PFS) was defined as the time interval from the 1st day of treatment to the date of objective tumor progression or death due to any cause, whichever occurred first. Statistical significance was determined at a level of *p* < 0.05.

## 4 Results

The demographic characteristics of all patients are shown in Table 1. The follow‐up period was 26 months for the 111 patients who received first‐line CDK4/6 inhibitor therapy. A total of 111 patients receiving first‐line CDK4/6 inhibitor therapy were included in the study. Of these, 62 patients (55.9%) were diagnosed with de novo metastatic disease. The remaining patients consisted of those with primary or secondary endocrine resistance and those with endocrine‐sensitive recurrent disease. The median age of the patients was 55 years (range, 23–82 years). The luminal A and B subgroups comprised 39 (35.1%) and 72 (64.9%) patients, respectively (Table 1).

**Table 1:** Demographic characteristics of patients.

Population of characteristics	*N* = 111 (%)
Age, years (Median) 55 (23–82)	
ECOG PS	
0	62 (55.9)
1–2	49 (44.1)
Histology at diagnosis	
Ductal	100 (90.1)
Lobular	10 (9.0)
Mix	1 (0.9)
SBR grade at diagnosis	
I	13 (11.7)
II	56 (50.5)
III	42 (37.8)
Stage of diagnosis	
II	24 (21.6)
III	25 (22.5)
IV	62 (55.9)
Menopause	
Yes	83 (74.8)
No	28 (25.2)
De novo metastatic cancer	
Yes	62 (55.9)
No	49 (44.1)
Previous treatment of non‐de novo disease	49 (44.1)
Adjuvant endocrine treatment	44 (89.7)
Neo‐adjuvant	3 (6.1)
Chemotherapy	1 (2.0)
No therapy	1 (2.0)
Recurrence during adjuvant endocrine treatment	
Yes	35 (79.5)
No	9 (20.5)
Early recurrence within the first 2 years	
Yes	13 (29.5)
No	31 (70.5)
Metastatic sites Visceral	26 (23.4)
Liver	13 (11.7)
Lung	18 (16.2)
Brain	2 (1.8)
Bone	86 (77.5)
Non‐regional lymph node metastases	45 (40.5)

The 44 of 49 (89.7%) patients with metastatic cancer had received adjuvant hormonal therapy for 5–10 years and/or four to six cycles of chemotherapy with adriamycin and taxane combinations. All 18 premenopausal patients received luteinizing hormone receptor agonists.

The follow‐up period was 26 months for the 111 patients who received first‐line CDK4/6 inhibitor therapy.

In total, 13 of 44 (29.5%), 22 (50%), and 9 (20.5%) patients receiving adjuvant or neoadjuvant therapy developed metastasis/recurrence at ≤ 24 months, 25–120 months, and > 120 months of hormonal treatment, respectively. Among these patients, 23 (20.7%) received palbociclib and hormonal therapy (an aromatase inhibitor and/or fulvestrant), and 88 (79.3%) received ribociclib and hormonal therapy (Table 1).

The numbers of patients with an NLR of < 2.40, PLR of < 153.6, and LMR of < 3.48 were 55 (49.5%), 55 (49.5%), and 54 (48.6%), respectively. The number of patients with an NLR, PLR, and LMR higher than these cut‐off values was 55 (50.5%), 56 (50.5%), and 57 (51.4%), respectively (Table 1).

Response distribution and best treatment in all patient groups are shown in Table 2.

**Table 2:** Treatment responses with CDK‐4/6i.

Population characteristics	
Median PFS (months)	26.0
Progression	No (%)
Yes	41 (36.9)
No	70 (63.1)
Died	13 (11.7)
Best response (in the first three months)	No. (%)
CR	22 (19.8)
PR	64 (57.7)
SD	19 (17.1)
PD	6 (5.4)

Abbreviations: CR, complete response; PD, progressive disease; PFS, Progressive‐free survival; PR, partial response; SD, stable disease.

Twenty‐two (19.8%) patients achieved a complete response, 64 (57.7%) patients achieved a partial response, 19 (17.1%) patients achieved stable disease, and 6 (5.4%) patients developed progressive disease in the first 3 months. Forty‐one (36.9%) patients receiving CDK4/6 inhibitors as first‐line treatment developed progressive disease. Thirteen (11.7%) patients died. The median PFS duration in these patients was 26 months, and the OS data were insufficient for statistical analysis.

Seventy‐two of 111 patients (64.9%) developed neutropenia. Twenty‐eight (38.9%) patients had grade 1 or 2 neutropenia, and 44 (61.1%) patients had grade 3 or 4 neutropenia. Dose reduction was performed in 16 (14.4%) patients, and the drug was discontinued in 2 (1.8%) patients due to side effects. The number of patients whose medication was interrupted due to side effects was 33 (29.7%). Other side effects were not evaluated due to incomplete data (Table 3).

**Table 3:** Distribution of neutropenia occurring with CDK‐4/6i treatment and dose adjustments of patients.

Safety	Distribution of subgroups no. (%)
Neutropenia	
Yes	72 (64.9)
No	39 (35.1)
Neutropenia grade	
I	8 (11.1)
II	20 (27.8)
III	37 (51.4)
IV	7 (9.7)
Dose reduction	
Yes	16 (14.4)
No	95 (85.6)
Interruptions in the dose of CDK4/6i	
Yes	33 (29.7)
No	78 (70.3)
Discontinuation of CDK4/6i due to adverse events	
Yes	2 (1.8)
No	109 (98.2)

Abbreviation: CDK4/6i, cyclin kinase 4/6 inhibitors.

**Figure 3:**
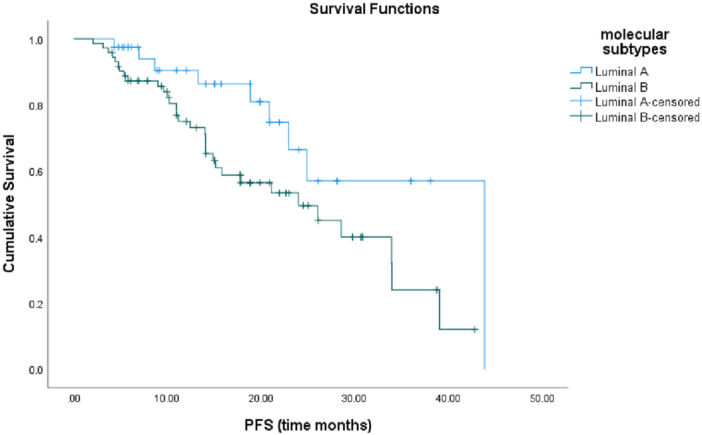
Progression‐free survival according to histologic subtypes.

According to univariate analysis, NLR, PLR, and LMR were not associated with tumor grade, subtypes, Ki‐67 index, stage, post‐menopausal status, side of metastases, preferred CDK4/6i and combined hormonal therapies, and neutropenia frequency. When evaluated in treatment subgroups, patients with serum LMR > 3.48 had better PFS compared to those with LMR < 3.48 (28.5 months and 13.2 months for palbociclib, respectively; Table 4, *p* = 0.04) (Figure 3).

**Table 4:** The impact of inflammatory markers on progression‐free survival according to treatment type.

Variables	Ribociclib		Palbociclib	*p‐*values**
Baseline NLR median = 2.40				
High	43.8	0.95	18.8	0.40
Low	39.0		23.9	
Baseline PLR median 153.6				
High	43.8	0.53	14.8	0.33
Low	39.0		23.9	
Baseline LMR median 3.48				
High	39.0	0.89	28.5	0.04*
Low	NR		13.2	

*Note:* Univariate analysis of factors associated with progression‐free survival. *p*‐values obtained from the log‐rank test. Cut‐off values for NLR, LMR, and PLR were determined by median split.

* Kaplan–Meier survival analysis

** Log‐rank Test *p* < 0.05 considered statistically significant.

**Figure 4:**
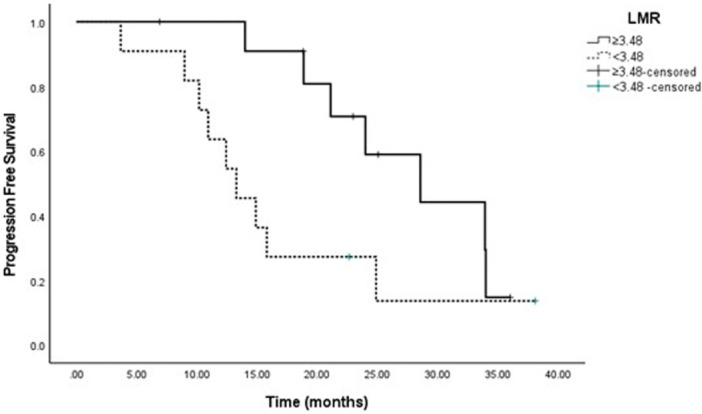
Palbociclib and ribociclib PFS graph according to LMR ≥ 3.48 and < 3.48 values.

Multivariate analysis was performed between variables with *p*‐values < 0.15 in the univariate analysis (Table 5). According to the multivariate analysis, worse PFS was observed in patients with molecular subtype luminal B (Table 5, *p* = 0.01) (Figure 4).

**Table 5:** Factors affecting prognosis in univariate and multivariate analysis (*n* = 111).

Variables	Subgroups No. (%)	Univariate analysis of PFS (months)*	*p‐*values**	Multivariate analysis *p‐*values*** ^‐^HR 95% CI
Age, years < 50 ≥ 50	43 (38.7) 68 (61.3)	33.9 23.9	0.41	
ECOG PS 0 I–II	62 (55.9) 49 (44.1)	23.9 26.0	0.45	
SBR grade at diagnosis I–II III	69 (62.2) 42 (37.8)	39.0 26.0	0.11	0.37, HR:1.3, 95% CI: 0.70–2.55
Menopause Yes No	83 (74.8) 28 (25.2)	26.0 33.9	0.82	
Ki‐67 ≥ %20 <%20	79 (71.2) 32 (28.8)	24.8 43.8	0.04	
*De novo* metastatic cancer Yes No	62 (55.9) 49 (44.1)	33.9 24.8	0.16	0.29, HR:1.4, 95% CI: 0.74–2.67
Visceral metastases Yes No	26 (23.4) 85 (76.6)	20.8 28.5	0.37	
Bone metastases Yes No	86 (77.5) 25 (22.5)	24.8 43.8	0.75	
CDK4/6i Ribociclib Palbociclib	88 (79.3) 23 (20.7)	39.0 21.0	0.08	0.16, HR:1.5, 95% CI: 0.83–3.02
Combined ET with CDK4/6i AI Fulvestrant	89 (80.2) 22 (19.8)	28.5 21.0	0.35	
Molecular subtypes of tumor Luminal A Luminal B	41 (36.9) 70 (63.1)	43.8 23.9	0.01	0.01, HR:2.7, 95% CI: 1.23–6.33
NLR (median = 2.40) High Low	56 (50.5) 55 (49.5)	33.9 26.0	0.87	
Lymphopenia (< 1.5 G/L) Yes No	40 (36.0) 71 (64.0)	33.9 26.0	0.72	
PLR (median = 153.6) High Low	56 (50.5) 55 (49.5)	24.8 26.0	0.51	
LMR (median = 3.48) High Low	57 (51.4) 54 (48.6)	33.9 22.9	0.22	
Neutropenia Yes No	72 (64.9) 39 (35.1)	26.0 39.0	0.76	

*Note:* Univariate analysis of factors associated with progression‐free survival. *p*‐values obtained from the log‐rank test. HR calculated from univariate Cox regression. Cut‐off values for NLR, LMR, and PLR were determined by median split.

Abbreviations: AI, aromatase inhibitors; CDK4/6i, cyclin kinase 4/6 inhibitors; Combined ET, combined endocrine treatment; ECOG PS, Eastern Cooperative Oncology Group Performance Status; SBR grade, Scarff‐Bloom Richardson; NR, non‐reached.

* Kaplan–Meier survival analysis.

** Log‐rank Test *p* < 0.05.

*** Cox‐regression analysis; HR, hazard ratio; 95%CI,confidence interval; *p* < 0.05 considered statistically significant.

## 5 Discussion

Peripheral blood neutrophil, lymphocyte, and monocyte counts, as well as derived indices such as the neutrophil‐to‐lymphocyte ratio (NLR), the platelet‐to‐lymphocyte ratio (PLR), and the lymphocyte‐to‐monocyte ratio (LMR), reflect the systemic inflammatory response and the tumor microenvironment [11].

In metastatic cancers, neutrophil precursors exhibit a different programming pattern, which also varies depending on the cancer microenvironment and the cytokines released. These immune modulatory cells can be reprogrammed by cancer therapy. They can be reprogrammed into pluripotent precursor cells, even if they are mature lymphocytes. These functional changes lead to different tumor responses [12, 13].

The effects of CDK4i therapy on peripheral blood cells may vary across different phases of the treatment course. Early during treatment, CDK4/6 inhibitors have been reported to induce transient changes in circulating leukocyte subsets, including a relative increase in neutrophil counts within the first days of therapy and a subsequent decline in lymphocyte counts by approximately the second week. An early increase in neutrophil count has been associated with poorer PFS [14].

Baseline leukocyte and neutrophil levels are not associated with progression‐free survival. High NLR levels tend to have shorter PFS and OS in patients with metastatic breast cancer [3, 15, 16, 17]. Early progression has been observed within 12 months in patients with an NLR of > 2.53 [3]. A high NLR (> 3) has been associated with poor ECOG performance status [18]. However, results vary according to molecular subtypes, and another study found that a low NLR and white blood cell count predicted worse disease‐free survival and higher mortality among patients with triple‐negative breast cancer [11, 15]. For patients with luminal A cancers, high LMR were favorable prognostic factors, while blood neutrophil, monocyte, and lymphocyte counts had no prognostic effect [19]. Among patients with luminal B cancers, low NLR and PLR had beneficial prognostic effects [15]. In our study, the NLR, PLR, and LMR cut‐off values were consistent with the general data. NLR, PLR, and LMR did not contribute significantly to PFS (*p* = 0.87, *p* = 0.51, *p* = 0.22, respectively). Multivariate analysis showed that the luminal B subgroup had 2.7 times worse PFS compared with the luminal A subgroup (hazard ratio: 2.7 [95% confidence interval: 1.23–6.33], *p* = 0.01). PFS was 23 months in luminal B and 43 months in luminal A.

PLR is another marker of inflammation and immune status. High PLR means worse survival outcomes; however, its value is less consistent than that of NLR. In one study, survival was better among patients with a PLR < 136.6, and early‐stage T1–T2 tumors compared to those with advanced‐stage tumors and metastatic disease (DFS: *p* = 0.032, OS: *p* = 0.012) [19].

An absolute lymphocyte count of ≥ 1.5 × 10⁹/L was associated with better PFS, compared to a lower lymphocyte count [20]. In one study, among patients with PR‐positive cancer, those with an absolute lymphocyte count < 1.50 × 10⁹ had better PFS [20]. Since anti‐cancer immunity is not dominant in patients with HR‐positive cancer, the absolute lymphocyte count is a less effective predictive factor [20, 21]. In our study, no contribution to PFS was observed in patients with lymphopenia < 1.5 g/L, LMR < 3.48, and neutropenia (*p* = 0.72, *p* = 0.22, *p* = 0.76, respectively). Treatment with CDK4/6i revealed heterogeneity in peripheral blood cells. When patients receiving ribociclib and palbociclib were evaluated separately, PFS was 39 months and 28.5 months, respectively, in the group with LMR > 3.48 (*p* = 0.04). In cancer immunotherapy, CD8⁺ cytotoxic T cells were reported to migrate to the tumor microenvironment to eliminate target tumor cells. However, in patients with high phosphoglycerate dehydrogenase (PHGDH) expression, the palbociclib‐induced increase in CD8⁺ T‐cell activity was shown to be attenuated, thereby reducing its therapeutic effect. This interaction was suggested to contribute to poorer clinical outcomes [22]. In several malignancies, including breast, colon, and melanoma, PHGDH was identified as a metabolic oncogene, and elevated levels of this enzyme were associated with adverse prognosis [23].

Grade 3–4 neutropenia related to ribociclib was detected in 60% of patients in the Monaleesa‐2, ‐3, and ‐7 studies. Therefore, the first drug dose was reduced in 30% of patients [24, 25, 26]. Neutropenia and dose reduction associated with palbociclib are more common, although they vary among ethnic groups (90% in Asians and 60% in non‐Asians, respectively, and 60% in Asians and 30% in non‐Asians) [27]. In the PALOMA‐2 study, neutropenia and the rate of dose reduction/discontinuation due to neutropenia was 66% and 25% [28]. In our study, the incidence of grade 3–4 neutropenia in patients receiving CDK4/6i was 61%, and the incidence of dose reduction/discontinuation was 16%. In these studies, overall response rates (ORR) were evaluated separately according to treatment arms and combination regimens, with reported ORRs of approximately 40% [24, 25, 27]. As our cohort was not suitable for similar stratification, the complete response rate was 19.8%.

In the our study, 6 (5.4%) of 111 patients developed progressive disease. These patients received chemotherapy or everolimus combined with exemestane, and they achieved a PFS of 26 months with first‐line CDK4/6 inhibitor treatment. Additionally, 13 (11.7%) patients died. The PFS durations with ribociclib in the MONALEESA‐2 trial were 25.3 and 27.6 months in the European Union and the United States, respectively [24, 29]. In the PALOMA‐2 trial, the PFS duration with palbociclib was 27.6 months [29].

## 6 Limitations

This retrospective study included a relatively small number of patients. Although patients with elevated C‐reactive protein (CRP) or procalcitonin levels were excluded, individuals with a prior history of elevated CRP were included if no infectious focus was identified and no treatment was administered. In our study, patients receiving different CDK4/6 inhibitors were evaluated as a single cohort because these agents share similar mechanisms of action. We also analyzed inflammatory responses collectively in pre‐, peri‐, and postmenopausal patient groups. Separate immune profiling for pre‐ and postmenopausal women was not performed, as menopausal status, as well as reproductive factors such as parity and breastfeeding history, may influence pro‐inflammatory signaling pathways.

## 7 Conclusion

Although a practical cut‐off value for peripheral blood inflammatory markers has not been established, these markers can determine the immune response of the host. In our study, no prognostic significance was shown for low and high cut‐off values of baseline NLR, PLR, and LMR, but PFS was better among patients with luminal A. In patients with LMR > 3.48, the ribociclib group demonstrated longer progression‐free survival compared with the palbociclib group.

## Author Contributions

**Nilufer Bulut:** writing – original draft, writing – review and editing. **Tanju Kapagan:** data curation. **Aykut Ozmen:** formal analysis, supervision. **Gokmen Umut Erdem:** formal analysis, supervision.

## Funding

The authors have nothing to report.

## Ethics Statement

The study was conducted according to the guidelines of the Nuremberg Code and Declaration of Helsinki and approved by the Ethics Committee of Basaksehir Cam and Sakura City Hospital (BSH‐2023‐182). Written informed consent was obtained from the patients.

## Consent for Publication

The authors have nothing to report.

## Conflicts of Interest

The authors declare no conflicts of interest.

## Data Availability

The data that support the findings of this study are available from the corresponding author upon reasonable request.
